# Pan-Genome-Based Characterization of the PYL Transcription Factor Family in *Populus*

**DOI:** 10.3390/plants14162541

**Published:** 2025-08-15

**Authors:** Xiaoli Han, Chen Qiu, Zhongshuai Gai, Juntuan Zhai, Jia Song, Jianhao Sun, Zhijun Li

**Affiliations:** 1 Xinjiang Production & Construction Corps Key Laboratory of Protection and Utilization of Biological Resources in Tarim Basin, Aral 843300, China; lilyan0509@163.com (X.H.); qiuchentea@163.com (C.Q.); gaizhongshuaitea@163.com (Z.G.); zhaijuntuan2022@163.com (J.Z.); nxy@163.com (J.S.); 2 College of Life Science and Technology, Tarim University, Aral 843300, China; 3 Desert Poplar Research Center, Tarim University, Aral 843300, China

**Keywords:** *Populus*, pan-genome, structural variation, *P. euphratica*, expression pattern, PYL

## Abstract

Abscisic acid (ABA) is a key phytohormone involved in regulating plant growth and responses to environmental stress. As receptors of ABA, pyrabactin resistance 1 (PYR)/PYR1-like (PYL) proteins play a central role in initiating ABA signal transduction. In this study, a total of 30 *PopPYL* genes were identified and classified into three sub-families (PYL I–III) in the pan-genome of 17 *Populus* species, through phylogenetic analysis. Among these subfamilies, the PYL I subfamily was the largest, comprising 21 members, whereas PYL III was the smallest, with only four members. To elucidate the evolutionary dynamics of these genes, we conducted synteny and *Ka*/*Ks* analyses. Results indicated that most *PopPYL* genes had undergone purifying selection (*Ka*/*Ks* < 1), while a few were subject to positive selection (*Ka*/*Ks* > 1). Promoter analysis revealed 258 *cis*-regulatory elements in the *PYL* genes of *Populus euphratica* (EUP) and *Populus pruinosa* (PRU), including 127 elements responsive to abiotic stress and 33 ABA-related elements. Furthermore, six structural variations (SVs) were detected in *PYL_EUP* genes and significantly influenced gene expression levels (*p* < 0.05). To further explore the functional roles of *PYL* genes, we analyzed tissue-specific expression profiles of 17 *PYL_EUP* genes under drought stress conditions. *PYL6_EUP* was predominantly expressed in roots, *PYL17_EUP* exhibited leaf-specific expression, and *PYL1_EUP* showed elevated expression in stems. These findings suggest that the drought response of *PYL_EUP* genes is tissue-specific. Overall, this study highlights the utility of pan-genomics in elucidating gene family evolution and suggests that *PYL_EUP* genes contribute to the regulation of drought stress responses in EUP, offering valuable genetic resources for functional characterization of *PYL* genes.

## 1. Introduction

Through evolutionary adaptation, plants have developed sophisticated regulatory networks to cope with abiotic stress [1,2,3,4], in which the phytohormone abscisic acid (ABA) plays a central role [5,6,7,8]. In plants, the ABA signaling cascade is initiated after ABA is perceived by pyrabactin resistance 1 (PYR1)/PYR1-like (PYL)/ABA receptors [9]. Under normal growth conditions, low ABA concentrations prevent PYR/PYL receptors from binding to PP2C phosphatases [10,11], thereby inhibiting *SnRK2* kinase activity and holding the ABA signaling pathway in an inactive state [12]. During drought stress, elevated ABA levels induce PYL expression and promote its interaction with PP2C, relieving the inhibition of *SnRK2* [13,14], activating downstream transcription factors and ion channels, and regulating stomatal closure and the expression of stress-responsive genes, ultimately enhancing plant drought resistance [15,16,17].

As members of the START protein superfamily, PYL proteins are characterized by a highly conserved START domain capable of binding hydrophobic ligands [18]. On the basis of this conserved gene sequence, 14 PYL genes containing the START domain have been identified in *Arabidopsis thaliana* and designated *AtPYR1–AtPYR13*. Phylogenetic analysis has classified these genes into three subfamilies [19,20]. Different subfamilies have been found to perform diverse functions in plant development and responses to abiotic stress. *AtPYL5* and *AtPYL9* have been identified as drought-resistant [21,22]. Under drought stress, *AtPYL5* has been shown to enhance the photosynthetic rate [23]; *AtPYR1*, *AtPYL2*, *AtPYL4,* and *AtPYL5* are involved in regulating stomatal and guard cell closure in response to CO_2_ [24]; and *AtPYL8* and *AtPYL9* play key roles in root growth and leaf senescence [25]. In addition, expression analysis of 11 ABA-related rate-limiting enzyme genes in different tissues of grapevine revealed that *VvPYL1* exhibits tissue-specific expression, and the highest expression level has been observed in roots [26]. As a core regulator of ABA signaling, the function of PYL has been elucidated in many species, including rice (*Oryza sativa*) [27], tomato (*Solanum lycopersicum*) [7], soybean (*Glycine max*) [28], wheat (*Triticum aestivum*) [29], maize *(Zea mays*) [30], poplar (*Populus*) [31], rubber tree (*Hevea brasiliensis*) [32], strawberry (*Fragaria × ananassa*) [33], cotton (*Gossypium hirsutum*) [34], and castor bean (*Ricinus communis*) [35]. These studies demonstrate that the *PYL* gene family, as central components of the ABA signaling pathway, considerably enhances plant adaptation to abiotic stresses by regulating diverse physiological processes, making their functional characterization crucial for elucidating ABA signal transduction mechanisms [36].

With the advancement of plant pan-genomics, cross-species comparative genomic analysis has become feasible. Compared with conventional gene family identification approaches, pan-genomes overcome the limitation of single reference genomes by enabling the detection of gene family members that are absent from the reference genome but present in other genomes. This advancement provides new opportunities to investigate gene family conservation and divergence, as well as their roles in adaptive evolution [37]. Based on the pan-genome of 26 high-quality maize genomes, Sun et al. identified extensive presence–absence variations and structural variations (SVs), thereby laying the foundation for investigating the TPS gene family and its biological functions [38]. In addition, Wang et al. [39] constructed a genus-level super-pan-genome comprising 19 *Populus* genomes, identifying 142,202 cross-species SVs that intersected with numerous genes and significantly contributed to phenotypic and adaptive divergence. This study also provided comprehensive multi-omics resources associated with the *Populus* super-pan-genome. Although the structure and function of *PYL* genes have been extensively characterized in the model plant *Arabidopsis thaliana* and various crop species, a systematic study of the *PYL* gene family has not yet been conducted within the genus *Populus* of the family Salicaceae. *Populus euphratica* (EUP), known for its exceptional environmental adaptability and recognized as the most stress-resistant representative of the *Turanga* section of the genus *Populus*, is widely distributed across Central Asia, West Asia, and the arid regions of northwest China [40,41]. Its unique physiological and ecological adaptations enable remarkable survival under extreme drought and saline-alkali conditions, establishing it as an ideal model species for studying abiotic stress responses in woody plants [42]. In this study, we conducted a comprehensive identification and analysis of the *PYL* gene family across 17 species of the genus *Populus*, encompassing the sections *Leucoides*, *Tacamahaca*, *Aigeiros*, *Turanga,* and *Leucoides*. We conducted a comprehensive analysis of the gene structure, conserved motifs, phylogenetic relationships, promoter elements, SVs, and gene expression patterns of *PYL* proteins. In addition, tissue-specific expression profiles of *PYL* members in EUP were precisely quantified using qRT-PCR technology. These findings not only provide a timely foundation for functional studies of the *PopPYL* gene family but also significantly enhance our understanding of abiotic stress adaptation mechanisms in EUP, while offering valuable candidate genes for future genetic engineering and breeding programs in this species.

## 2. Results

### 2.1. Pan-Genome Distribution of the PopPYL Gene Family

Due to the limitations of a single reference genome in capturing the genetic diversity of a species, we collected 17 high-quality poplar genomes (Table 1) and conducted a pan-genome-level analysis of *PYL* genes to better understand their genetic variation. First, a comprehensive genome-wide comparative analysis of the *PYL* gene family was performed using BLASTP and HMM-based methods, resulting in the identification of 352 *PYL* genes in total. The number of *PYL* genes in each poplar genome ranged from 18 to 22 (Table S1). Orthologous genes across the 17 genomes were identified using OrthoFinder (v2.5.2; default parameters). The pan-gene IDs of the *PYL* gene family were extracted from the “Pan-genes.list” output file (Table S2), and genes sharing the same pan-gene ID across species were considered the same gene. Ultimately, 30 *PYL* pan-genes were obtained. Among these, 20 *PopPYL* genes were identified in *Populus yunnanensis*, *Populus wuana* (WUA), *Populus trichocarpa* (TRI), *Populus simonii* (SIM), and *Populus lasiocarpa*. Additionally, 18 *PopPYL* genes were detected in *Populus tremula* (TRE), *Populus szechuanica* (SZE), *Populus pseudoglauca* (PSE), *Populus koreana*, *Populus davidiana*, *Populus alba* var. *pyramidalis* (ALB), *Populus adenopoda* (ADE), *Populus rotundifolia*, and *Populus qiongdaoensis* (QIO). Notably, EUP (Table S3) and *Populus pruinosa* (PRU) exhibited the lowest number of *PYL* genes, with only 17 identified in each, whereas *Populus deltoides* (DEL) contained the highest number, with a total of 22 *PYL* genes.

Notably, QIO contained the unique genes *Qdy09060.t1* (*PYL3*) and *Qdy37849.t2* (*PYL26*), whereas ALB harbored three unique genes: *pal_pou08344.t1* (*PYL8*), *pal_pou07031.t1* (*PYL21*), and *pal_pou33677.t1* (*PYL22*). Similarly, PRU possessed *PprTF01G0764.1* (*PYL23*), ADE contained *Poade06008.t2* (*PYL24*) and *Poade13605.t2* (*PYL25*), SIM carried *Posim17381.t2* (*PYL27*) and *Posim35007.t2* (*PYL28*), TRE carried *Potra2n8c17195.8* (*PYL29*), and WUA maintained *Powua15969.t2* (*PYL30*). These species-specific genes may be associated with adaptive evolution in their respective species. Conversely, multiple *PYL* genes demonstrated high conservation across all genomes. Core *PYL* genes, including *PYL1*, *PYL2*, *PYL4–7,* and *PYL9–17*, were universally present in all 17 examined genomes. These conserved genes likely play essential roles in the fundamental physiological processes of *Populus* species and have remained evolutionarily stable.

Phylogenetic analysis of the *Populus PYL* protein family revealed three distinct subfamilies (I, II, and III), which exhibited significant divergence in their physicochemical properties, including amino acid length, molecular weight, isoelectric point (pI), instability index, and aliphatic index (Table 2). Subfamily I members displayed the greatest variability, with the amino acid length ranging markedly from 54 residues to 706 residues. *PYL3* (706 amino acids) and *PYL8* (615 amino acids) represented the largest proteins within this subfamily, whereas *PYL18* (102–115 amino acids) was the smallest. This subfamily exhibited the broadest molecular weight distribution (9.7–77.3 kDa) and predominantly acidic isoelectric points (pI 4.4–10.2), and *PYL21* (pI 8.44) was the sole alkaline exception. In contrast, subfamily II proteins exhibited a more conserved amino acid length (186–306 amino acids) and molecular weight (20.6–34.1 kDa), but were distinguished by their markedly alkaline isoelectric points (pI 7.1–9.2), a feature potentially related to their subcellular localization or functional specialization. The protein length of subfamily III members ranged from 169 amino acids (*PYL20*) to 201–259 amino acids (*PYL7*), with the molecular weight spanning 13.5–28.7 kDa. Notably, *PYL7* exhibited the highest molecular weight within this subfamily (22.4–28.7 kDa). All members were characterized as acidic proteins (pI 4.93–7.01), and *PYL9* showed the strongest acidity (pI 4.93–5.53). Hydrophilicity analysis (GRAVY index 0.29–0.52) confirmed their hydrophilic nature, among which *PYL5* demonstrated the highest hydrophilicity.

### 2.2. Construction of the Phylogenetic Tree of PopPYL Gene Family Members

**Figure 1:**
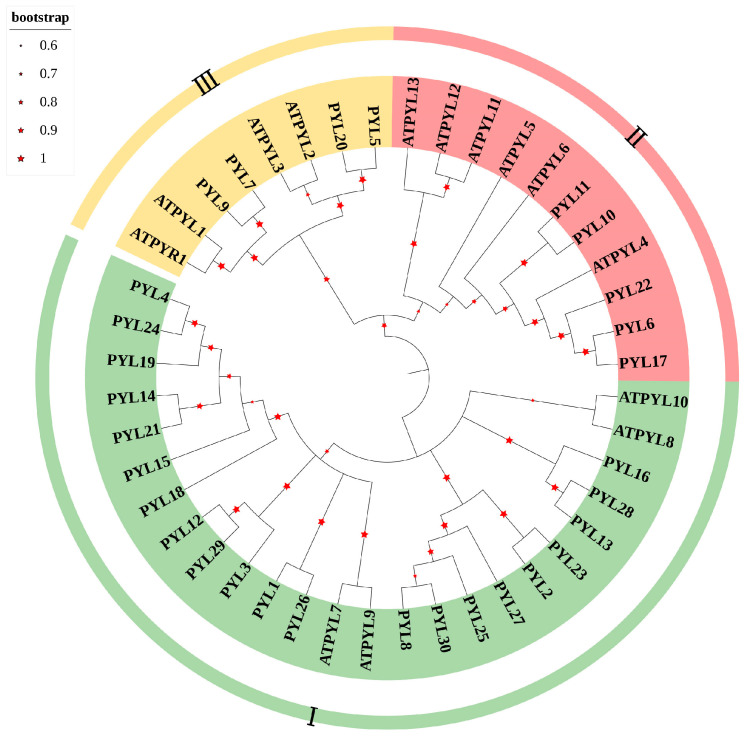
Phylogenetic analysis of *PYL* genes in *Populus*. An unrooted Neighbor-Joining phylogenetic tree was constructed from *Arabidopsis* and 30 *PopPYL*. The bootstrap test was set to 1000 replicates. Different colors represent different subfamilies of the *PYL* gene.

A phylogenetic tree was constructed based on the amino acid sequences of 14 *AtPYL* (*Arabidopsis thaliana*) and 30 *PopPYL* genes, revealing that the *PopPYL* proteins can be classified into three clades (PYL I, PYL II, and PYL III) following the established classification system for *AtPYL*, consistent with previous findings [51]. As shown in Figure 1, the PYL I subfamily was the most abundant, comprising 21 genes, followed by PYL II with 5 members, while PYL III was the smallest subfamily, containing only 4 members. This numerical disparity may reflect varying selective pressures and functional demands during the evolutionary diversification of these subfamilies. Within the PYL I subfamily, SZE uniquely possessed three copies of *PYL13*, whereas all other species retained only a single orthologous gene. Notably, *PYL3* and *PYL8* were detected exclusively in QIO and ALB, respectively, while *PYL21–PYL30* exhibited single-species-specific distribution patterns. Additionally, EUP and PRU exhibited the loss of *PYL15*, although PRU uniquely harbored a distinct *PYL23* gene. In the PYL II subfamily, *PYL22* was identified as a species-specific gene in ALB, whereas *PYL6*, *PYL10,* and *PYL11* were conserved as single-copy genes across all examined species. In contrast, *PYL17* was absent in ALB, ADE, and QIO. For the PYL III subfamily, *PYL7* and *PYL9* were conserved as single copies across all 17 species, while *PYL20* exhibited species specificity, being present only in WUA and PSE. The differential distribution of subfamily members across species suggests functional divergence within the *PYL* gene family, potentially linked to species-specific adaptations.

### 2.3. Chromosomal Location of PopPYL Genes in There Populus Species

**Figure 2:**
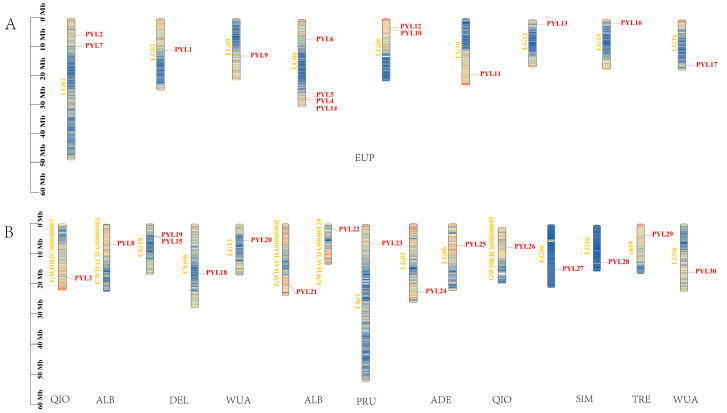
Distribution of the *PYL* genes in the reference genome (**A**) and non-reference genome (**B**) genome. PYL21-PYL30 were species-specific genes. The left axis shows the length of each chromosome and contig, as was estimated in megabases (Mb).

*Populus* is a diploid species (2n = 18) with 18 chromosomes. To investigate the chromosomal distribution characteristics of *PYL* genes in poplar, we performed chromosomal localization analysis of 30 *PopPYL* genes (Figure 2). The results revealed both conserved features and species-specific variations in their distribution patterns. Overall, *PYL* genes were dispersed across multiple chromosomes without concentrated distribution on any single chromosome, reflecting genomic structural differences and evolutionary divergence among species. In the reference genome, we identified the following *PYL* genes: *PYL1*, *PYL2*, *PYL4*, *PYL5*, *PYL6*, *PYL7*, *PYL9*, *PYL10*, *PYL11*, *PYL12*, *PYL13*, *PYL14*, *PYL16*, and *PYL17*. Among these, *PYL1*, *PYL2*, *PYL5*, *PYL6*, *PYL7*, *PYL9*, *PYL10*, and *PYL11* were conserved across all 17 *Populus* genomes, while *PYL4*, *PYL12*, *PYL13*, *PYL14*, *PYL16*, and *PYL17* were present only in specific poplar species (Figure 2A). Additionally, 16 species-specific *PYL* genes were distributed across non-reference genomes (Figure 2B), with QIO, ALB, DEL, WUA, SIM, ADE, TRE, and PRU containing 2, 3, 3, 2, 2, 2, 1, and 1 gene, respectively. These genes compensate for the limitations of identifying gene family members using a single reference genome. In conclusion, the 30 homologous *PopPYL* genes collectively represent the *PYL* pan-genes across the 17 *Populus* species.

### 2.4. Gene Structure and Conserved Motif Analysis

**Figure 3:**
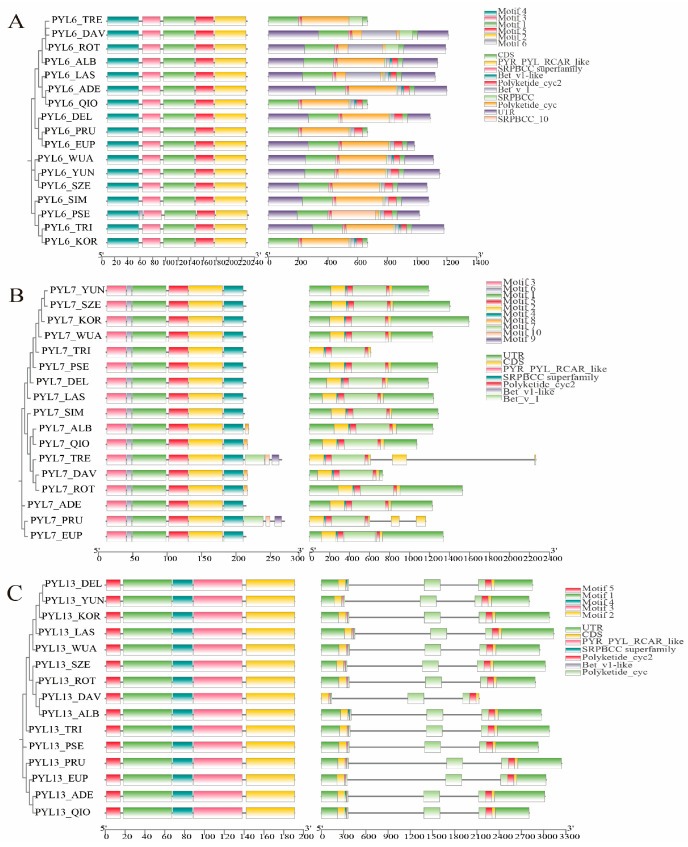
Comparison of the conserved motifs and gene structures of intermediate clade genes *PopPYL6* (**A**), *PopPYL7* (**B**), and *PopPYL13* (**C**) in 17 *Populus*. Left panel: the phylogenetic tree was constructed with the full length of PYL proteins using MEGA 7.0. Middle panel: the conserved motifs for PYL proteins. Motifs 1–10 are highlighted with different colors. Right panel: exon–intron structure of *PYL* genes.

Based on the evolutionary relationships between *PopPYL* and *AtPYL* genes, we analyzed the exon–intron structures of *PYL6*, *PYL7,* and *PYL13*, which are homologous to *AtPYL5*, *AtPYR1,* and *AtPYL8*, respectively, in 17 *Populus* species using TBtools software (v2.012). The results (Figure 3) revealed that *PYL6* genes lacked introns in all 17 *Populus* species, while *PYL13* genes consistently contained two introns. Members with identical or similar protein lengths shared conserved exon–intron structures, whereas genes with significant variation in protein length exhibited distinct gene architectures. For example, both *PYL7_TRE* and *PYL7_PRU*, which encode proteins of 255 and 259 amino acids, respectively, possessed two introns, whereas other *Populus PYL7* genes encoding shorter proteins (201–207 amino acids) exhibited no detectable introns. This structural diversity may reflect species-specific physiological functions and environmental adaptations. Notably, *PYL6*, *PYL7,* and *PYL13* genes all contained two exons across species, and homologous *PYL* genes maintained similar exon–intron patterns, further supporting their close evolutionary relationships and validating the current subfamily classification.

The conserved motifs of *PYL6*, *PYL7*, and *PYL13* genes across 17 *Populus* species were further analyzed. *PYL6* and *PYL13* members exhibited highly similar motif types and arrangements, and all the members contained five conserved motifs (*motif1–5*), except *PYL6_PSE*, which harbored an additional motif (motif6; Figure 3). In contrast, *PYL7* genes displayed considerable diversity in motif composition, and the number of motifs ranged from six to nine across species. Notably, *motif1–5* was universally present in all *PYL* proteins, indicating a high degree of evolutionary conservation. Moreover, *PopPYL* genes with varying protein lengths and gene structures showed distinct motif patterns. For example, within the *PYL7* subfamily, *PYL7_TRE* and *PYL7_PRU* exhibited longer protein lengths, divergent gene structures, and a greater number of conserved motifs compared to other *Populus* species. These findings suggest that *PopPYL* members within the same subfamily share conserved motif characteristics, supporting functional conservation, while the presence of unique motifs may confer specialized biological functions.

### 2.5. Synteny Analysis of PopPYL Genes

**Figure 4:**
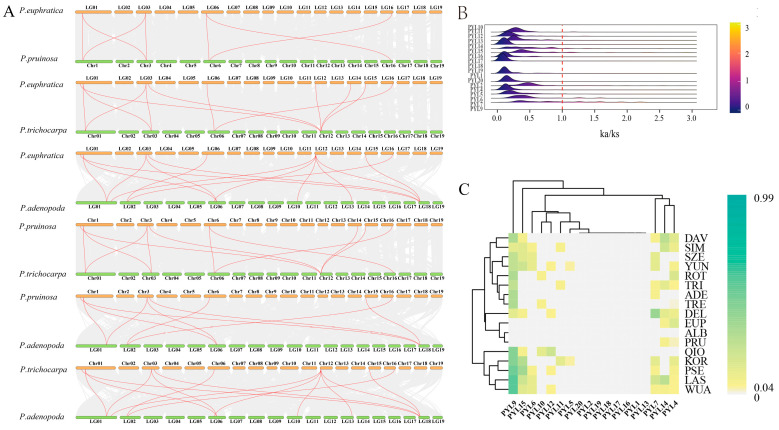
*Ka*/*Ks* values of *PopPYL*. (**A**) Synteny analysis of PYL among *P. euphratica* and other plant species. (**B**) Distribution of *Ka*/*Ks* values of *PopPYL* in 17 *Populus* varieties. (**C**) Heatmap of the frequency of occurrence of different *Populus* varieties at each *PYL* with a *Ka*/*Ks* ratio > 1.

Genome-wide synteny analysis of *PYL6*, *PYL7*, *PYL9,* and *PYL13* genes among four *Populus* species (EUP, TRI, PRU, and ADE) identified 61 syntenic gene pairs. Specifically, 14 homologous gene pairs were observed between TRI and ADE, while 11 pairs were detected between EUP and TRI, as well as between TRI and PRU. Additionally, 10 homologous pairs were identified between EUP and ADE, 9 between PRU and ADE, and 6 between EUP and PRU (Figure 4A). These findings suggest that the evolution and expansion of these genes primarily resulted from whole-genome duplication events.

Further *Ka*/*Ks* analysis of the *PYL* gene family across 17 *Populus* species revealed that most gene pairs exhibited *Ka*/*Ks* ratios significantly less than 1, indicating strong purifying selection during evolution (Figure 4B). This conservation of key functional domains is likely associated with their essential roles in plant stress adaptation. However, certain gene pairs, including *PYL4*, *PYL5*, *PYL6*, *PYL7*, *PYL9*, *PYL10*, *PYL11*, *PYL12*, *PYL14*, *PYL15*, and *PYL19*, displayed *Ka*/*Ks* values greater than 1, suggesting potential positive selection in some *Populus* lineages (Figure 4C). Notably, *PYL4*, *PYL6*, *PYL7*, *PYL9*, *PYL14*, and *PYL15* frequently exhibited *Ka*/*Ks* > 1 across multiple species, implying strong positive selection pressure during *Populus* evolution. In contrast, *PYL5*, *PYL10*, *PYL11*, and *PYL12* appeared to undergo positive selection only in specific species.

### 2.6. Promoter Analysis of PopPYL Genes

**Figure 5:**
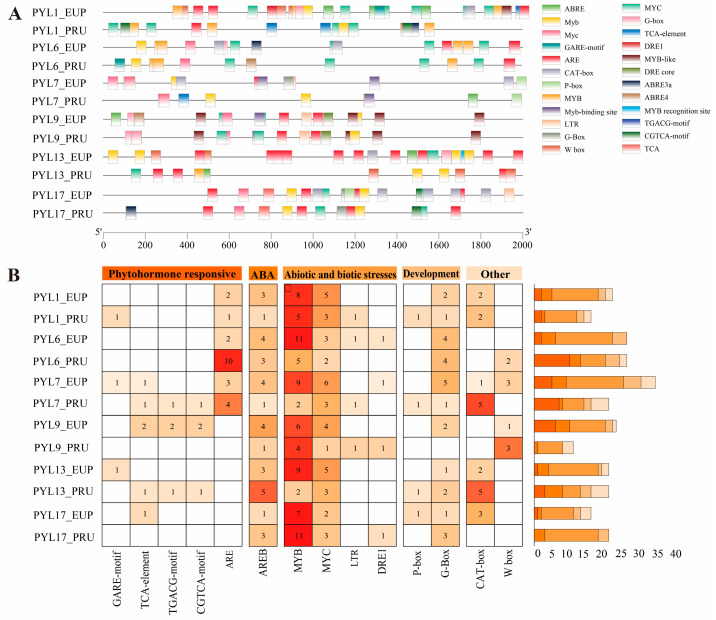
(**A**) Predicted cis-elements in promoter regions of *PYL_EUP* and *PYL_PRU* genes. (**B**) Cis-regulatory elements in PYL promoters of EUP and PRU. The depth of red square indicates the number of cis-acting elements for each gene, while white square indicates 0.

To elucidate the transcriptional regulatory mechanisms of *PYL_EUP* and *PYL_PRU*, a comparative analysis and PlantCARE-based screening of their promoter regions (2 kb upstream of the start codon) was performed (Figure 5A). A total of 258 *cis*-regulatory elements were identified and classified into five functional categories: plant growth and development (29 elements), abiotic stress response (127 elements), hormone signaling (39 elements), ABA-responsive elements (33 elements), and light response (30 elements). Notably, the AREB elements (ABA-responsive) were ubiquitously present, the highest abundance (five elements) was observed in *PYL17_PRU,* and only one was detected in *PYL7_EUP*/*PRU* (Figure 5B). Among abiotic stress-responsive elements, MYB elements associated with environmental adaptation exhibited significant variation in distribution. *PYL17*_*EUP*/*PRU* contained the fewest (2 elements), whereas *PYL9*_*EUP*/*PRU* exhibited the highest number (11 elements). Additionally, long terminal repeat (LTR) retrotransposon sequences were identified in *PYL7*/*9*/*13*/*17_EUP*. Hormone-responsive elements showed distinct patterns: auxin response elements (AREs) were widely distributed, with maximum enrichment in *PYL13*_*EUP* (ten elements) and a minimum of one element in *PYL7_EUP* (exclusively present in *P. euphratica*). JA/MeJA-responsive elements (TGACG motif and CGTCA motif) were restricted to *PYL17*_*EUP*/*PRU* and *PYL1*_*PRU* (≥1 element each), while SA-responsive (TCA element) and gibberellin-responsive elements (GARE motif) were sparsely distributed. Growth- and development-related CAT-box elements exhibited a broad distribution, peaking in *PYL17*_*EUP*/*PRU* (five elements) and with the lowest abundance in *PYL1_EUP* (one element, specific to EUP). Light-responsive G-box elements were found in all genes except *PYL13_PRU*, with maximum enrichment in *PYL1*_*EUP*/*PRU* (five elements). P-box elements were exclusively detected in *PYL7*_*EUP*/*PRU* and *PYL17*_*EUP*/*PRU*.

### 2.7. Structural Variation Analysis of PYL_EUP Genes

**Figure 6:**
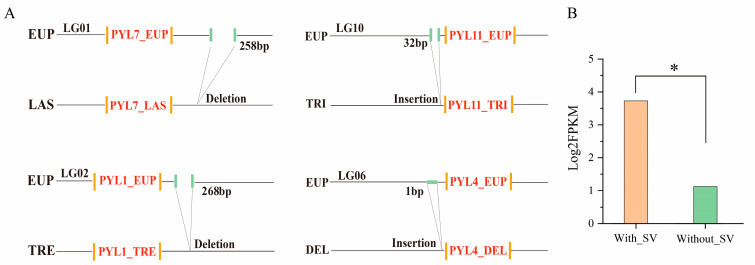
Effect of SVs on genes. (**A**) The effects of SV insertion and deletion on *PYL_EUP*. (**B**) The expression of *PYL*_*EUP* was significantly affected by SVs. (* *p *< 0.05).

Structural variations (SVs), commonly defined as DNA sequence alterations exceeding 50 base pairs (bp) in length, encompass a range of mutation types including insertions (INS), deletions (DEL), inversions, translocations, and copy number variations. These genomic alterations can directly affect protein function or gene expression by modifying coding sequences, gene copy numbers, or regulatory regions, thereby contributing to species evolution and phenotypic diversity. In the present study, a pan-genome was constructed using *P. euphratica* (EUP) as the reference genome, and SV analysis was conducted across 17 *Populus* species. Within the *PYL* gene family of *P. euphratica*, six structural variation sites were identified, primarily located in upstream, downstream, and intronic regions, comprising two insertions and four deletions (Figure 6). Specifically, one insertion (562 bp) was located upstream of *Peu06G022430* on LG06, and another (32 bp) was found upstream of *Peu10G016340* on LG10, both potentially introducing novel *cis*-regulatory elements. Meanwhile, a 258 bp deletion occurred 459 bp downstream of *Peu01G012370* on LG01, and a 268 bp deletion occurred 271 bp downstream of *Peu02G014980* on LG02, which may attenuate transcriptional regulation of these genes. In addition, intronic regions of *Peu15G001800* and *Peu02G014980* harbored a 1 bp insertion and a 725 bp deletion, respectively. These variations can disrupt mRNA splicing efficiency and result in aberrant transcripts. To evaluate the functional impact of these SVs, we employed Mann–Whitney U tests to compare expression levels between PYL genes with and without SVs. The results revealed statistically significant differences (*p* < 0.05), suggesting that structural variations substantially influenced the expression patterns of *PYL_EUP* genes. Collectively, these findings provided novel molecular insights into the evolutionary dynamics and functional diversification of the *PYL* gene family in *Populus*.

### 2.8. Expression Analysis of the PYL_EUP Gene Family

**Figure 7:**
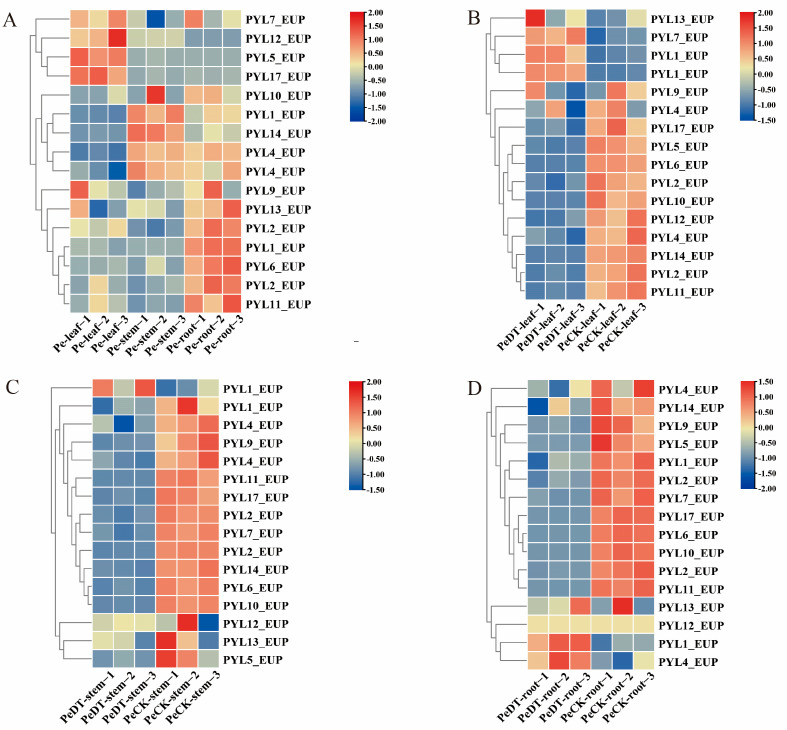
The expression pattern of *PYL* genes under drought stress was analyzed by heat mapping. (**A**) Pe-leaf vs. Pe-stem vs. Pe-root; (**B**) PeDT-leaf vs. PeCK-leaf; (**C**) PeDT-stem vs. PeCK-stem; (**D**) PeDT-root vs. PeCK-root.

Abscisic acid (ABA), a key phytohormone involved in regulating plant growth and stress responses, initiates its signaling pathway through recognition by the Pyrabactin Resistance 1-like (PYR/PYL) receptor family. Based on RNA-seq data analysis of *P. euphratica* under drought stress (Table S4), 16 *PYL_EUP* genes, excluding *PYL16_EUP*, exhibited differential expression patterns across root, stem, and leaf tissues (Figure 7A). Notably, *PYL1*/*2*/*6*/*9*/*11*/*13*_*EUP* were predominantly expressed in roots, suggesting their involvement in root-cap ABA signaling transduction. In contrast, *PYL5*/*7*/*12*/*17*_*EUP* showed leaf-specific upregulation, indicating potential roles in stomatal closure or leaf developmental processes. Meanwhile, the stem-preferential expression of *PYL1*/*4*/*10*/*14*_*EUP* implied a regulatory function in coordinating whole-plant drought responses, possibly by mediating ABA long-distance transport through phloem or xylem vascular tissues. This organ-specific divergence in expression clearly demonstrated the functional specialization of *PYL_EUP* family members across different tissues of *P. euphratica*, thereby optimizing ABA-mediated adaptation to drought stress.

To elucidate the potential roles of *PYL*_*EUP* genes in response to drought stress, their expression patterns were analyzed across different seedling tissues subjected to drought treatment. Heatmap analysis revealed distinct tissue-specific expression profiles among *PYL* family members under drought conditions (Figure 7B–D). These patterns suggest a functional differentiation of *PYL_EUP* genes in mediating ABA signaling and stress adaptation in a tissue-dependent manner. In leaves, *PYL1*_*EUP*, *PYL7*_*EUP*, and *PYL13*_*EUP* were upregulated under drought conditions, whereas *PYL2*/*4*/*5*/*6*/*10*/*11*/*12*/*14*/*17*_*EUP* exhibited downregulated expression (Figure 7B). In stems, only *PYL1*_*EUP* showed increased expression during drought stress, while all other *PYL_EUP* genes were downregulated (Figure 7C). In roots, *PYL1_EUP*, *PYL4_EUP*, *PYL12_EUP,* and *PYL13_EUP* were upregulated, and the remaining genes showed reduced expression levels (Figure 7D). Notably, *PYL1*_*EUP* demonstrated consistent upregulation in roots, stems, and leaves, highlighting its potential central role in the core regulatory network of the drought stress response in EUP. These results suggest that different *PYL_EUP* genes may contribute to drought adaptation through tissue-specific and functionally distinct regulatory mechanisms.

### 2.9. Verification of PYL Gene Expression Patterns in Different Tissues by RT-qPCR

**Figure 8:**
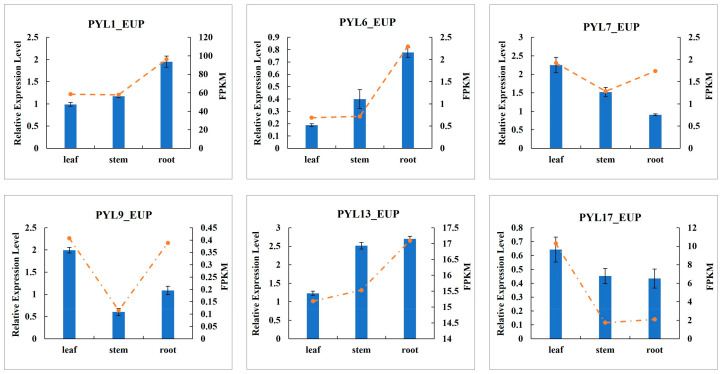
Expression of *PYL* genes analyzed by RT-qPCR in different tissues of *P. euphratica*. The bars are standard deviations (SDs) of three biological replicates.

To further elucidate the functional characteristics of the *PYL_EUP* gene family under drought stress, we performed a comparative expression analysis of six *PYL_EUP* genes across different plant tissues using quantitative real-time PCR (qRT-PCR). The results revealed significant tissue-specific expression differences among the selected *PYL* genes (Figure 8). Notably, *PYL7_EUP*, *PYL9_EUP,* and *PYL17_EUP* exhibited relatively higher expression levels in seedling leaves. In particular, *PYL7_EUP* showed peak expression in leaves and moderate expression in stems, whereas *PYL17_EUP*, although little expressed overall, displayed distinct preferential expression in leaf tissue. Conversely, *PYL13_EUP* was predominantly expressed in seedling stems. Additionally, *PYL1_EUP*, *PYL6_EUP,* and *PYL13_EUP* were primarily expressed in roots, and *PYL1_EUP* exhibited markedly higher expression in roots than in other tissues. *PYL6_EUP* demonstrated a graded expression pattern across organs, while *PYL13_EUP* showed its highest expression in root tissue. Validation of gene expression patterns demonstrated a strong correlation between qRT-PCR (2^–ΔΔCt^) and RNA-seq (FPKM) data for most genes (*PYL1*, *PYL6*, *PYL7*, *PYL13,* and *PYL17*). These findings underscore the functional diversification of *PYL* family members during drought adaptation and provide valuable molecular insights for future mechanistic studies.

## 3. Discussion

In this study, a high-quality pan-genome was constructed based on 17 *Populus* species, overcoming the limitations associated with relying on a single reference genome and enabling the comprehensive identification of full-spectrum genetic variations, including non-reference genes. By aligning 14 *Arabidopsis AtPYL* protein sequences, a total of 30 *Populus PYL* genes were identified in the pan-genome, comprising 17 reference-based genes and 13 non-reference genes. Among these, DEL possessed the highest number of *PYL* genes (22), while EUP and PRU exhibited the lowest gene counts (17). This variation in gene number may reflect divergent evolutionary trajectories among *Populus* species, and certain lineages underwent multiple gene duplication events [39] that contributed to the expansion of the *PYL* gene family. However, the duplicated genes have been subject to species-specific selective pressures, resulting in different evolutionary outcomes. Some *PYL* genes were conserved across most species due to essential functional roles, while others experienced functional divergence or were lost entirely in certain lineages. For instance, the eight *PopPYL* genes present in all species likely play central and conserved roles in fundamental biological processes, such as plant hormone signaling, cell growth, and development. In contrast, the 12 *PopPYL* genes unique to individual species might contribute to species-specific ecological adaptations or physiological functions, potentially aiding in responses to environmental stresses or specialized growth and developmental processes.

This study investigated the structural characteristics of *PYL6*, *PYL7,* and *PYL13* genes across 17 *Populus* species, revealing potential functional divergence within this gene family. The results showed that *PYL13* consistently contained two introns in all species, maintaining a highly conserved intron–exon structure across *Populus*. In contrast, *PYL7* exhibited substantial SV. Only *PYL7_TRE* and *PYL7_PRU* possessed two introns, whereas other members were intronless. This divergence may reflect functional differentiation following gene duplication, paralleling observations in *Arabidopsis*, where *AtPYL1–AtPYL8* typically contain 1–2 introns, while *AtPYL9–AtPYL13* are predominantly intronless, suggesting distinct evolutionary trajectories among subfamilies [52]. Motif analysis revealed that motifs 1–5 were universally present across all *PYL* proteins, likely corresponding to core functional domains of START-like ABA receptors, consistent with conserved ABA-binding sites reported in *Arabidopsis PYL* proteins [20]. However, *PYL7* members displayed notable diversity in motif composition (6–9 motifs), particularly due to the presence of unique additional motifs in *PYL7_TRE* and *PYL7_PRU*, which were associated with their extended protein length. Similar patterns were observed in the tomato *SlPYL* family, where C-terminal extensions conferred regulatory specificity to certain members [53]. These variations may reflect adaptive responses to drought or cold stress. Furthermore, significant differences in protein length and motif organization among *PYL7* members (e.g., *PYL7_TRE* with 259 amino acids versus 201–207 in others) suggest potential subfunctionalization or neofunctionalization. For instance, in maize, *ZmPYL10* acquired distinct ABA sensitivity through the insertion of a specific sequence [54].

Gene family expansion in plant evolution is primarily driven by whole-genome duplication (WGD) and tandem duplication [55]. Our multiple synteny analysis of four core *Populus PYL* genes revealed extensive syntenic relationships across species, consistent with the predominant mechanisms of plant gene family expansion. Previous studies have shown that the genus *Populus* underwent a unique WGD event, namely, the Salicoid duplication, approximately 60 million years ago, which contributed to the expansion of numerous gene families, including those involved in ABA signaling pathways [56]. Selection pressure analysis revealed that the majority of *PYL* gene pairs exhibited *Ka*/*Ks* ratios significantly less than 1 (*p* < 0.01), indicating strong purifying selection during evolution. This likely preserved their conserved functions in ABA perception and signal transduction. These findings are consistent with previous studies on the rice *OsPYL* family, in which the START-like domain of ABA receptor proteins demonstrated high conservation [57]. However, *PYL4*, *PYL6*, *PYL7*, *PYL9*, *PYL14,* and *PYL15* frequently showed *Ka*/*Ks* > 1 across multiple *Populus* species, suggesting that these genes may confer enhanced adaptive advantages under specific environmental stresses, such as drought or salinity. Similarly, the positive selection signals observed in *PYL6* and *PYL9* may reflect species-specific adaptation strategies to abiotic stresses, analogous to the drought tolerance conferred by positive selection in maize *ZmPYL10* [54].

Insertion and deletion (InDel) variations in genomes can directly alter gene coding sequences, resulting in amino acid changes or frameshift mutations that ultimately affect protein function [58]. For example, in peanut (*Arachis hypogaea*), an insertion variant in the 3′-UTR of *AhCKX6* reduced gene expression and increased active cytokinin levels, thereby promoting grain enlargement [59]. Similarly, a 358 bp insertion in the promoter region of maize *bZIP68* significantly upregulated its expression [60]. Structural variations (SVs) also play crucial roles in plant stress adaptation and evolutionary processes. In rice, for instance, long SVs are significantly enriched in promoter regions, and strong colocalization was observed between SVs and stress-responsive genes, suggesting that SVs contribute to the pleiotropic expression patterns commonly observed in stress-related genes [61]. In our investigation of the *PYL* gene family in EUP, we identified six SVs (two insertions and four deletions) predominantly located in regulatory regions. These included 1 bp and 32 bp insertions upstream, and 258 bp and 268 bp deletions downstream of *PYL* genes. These SVs may influence gene expression by modifying regulatory sequences or interfering with splicing, thus modulating ABA signaling pathways and environmental adaptation. Notably, SVs within intronic regions (a 1 bp insertion and a 725 bp deletion) can alter the spatial configuration of splice donor or acceptor sites, potentially increasing alternative splicing events. As demonstrated in rice *Ptr* gene studies, atypical gene structures induced by SVs (including variations in exon number or protein truncation) may still retain essential biological functions [62].

*cis*-acting elements in plant *PYL* genes play pivotal roles in environmental stress responses and signaling pathways that govern plant growth and development [36]. These regulatory elements can be classified into three major categories: hormone-responsive elements (e.g., ABRE) [63], growth-related elements (e.g., CAT-box) [64], and stress-responsive elements (e.g., MBS) [12]. Our systematic analysis of promoter regions (2 kb upstream) in *PYL* gene families from EUP and PRU identified 64 distinct *cis*-acting elements, encompassing light-responsive, abiotic stress-related, and hormone-responsive elements. As crucial components of ABA signaling, *PYL* gene promoters are particularly enriched with AREB elements, and *PYL17*_*PRU* exhibits the highest abundance. These findings suggest *PYL17*_*PRU*’s potential significance in ABA-mediated stress responses [65]. Notably, ARE elements involved in phytohormone responses exhibit substantial distribution variation, being exclusively present in EUP, with maximal enrichment in *PYL13*_*EUP*. Among abiotic stress-related elements, MYB transcription factors, which regulate plant cell morphogenesis [66], developmental processes [67], and stress adaptation [68], showed marked distribution variation. *PYL9*_*EUP*/*PRU* demonstrated the highest density, indicating its enhanced environmental stress sensitivity. Furthermore, JA/MeJA-responsive elements were uniquely distributed in *PYL17_EUP*/*PRU* and *PYL1_PRU*, implying their potential involvement in jasmonate signaling-mediated stress resistance. The distinct distribution patterns of these *cis*-elements reveal the sophisticated transcriptional regulatory mechanisms underlying the multifunctional roles of *PYL* genes in plant development, hormone signaling, and stress adaptation.

Gene expression patterns are closely related to biological functions. This study systematically analyzed the tissue-specific expression and drought stress response characteristics of the *PYL_EUP* gene family in EUP, providing insights into its physiological roles and regulatory mechanisms. Previous studies have demonstrated that plant *PYL* gene family members generally exhibit tissue-specific expression patterns. In soybean seeds, most *PYL* genes showed significantly higher expression levels than in other tissues [28]. During oilseed germination, *PYL* genes displayed high transcriptional abundance [69]. In rubber tree latex, *PYL* gene transcripts were particularly abundant [32]. Additionally, *PYL* genes exhibited predominant expression in Chinese cabbage callus [70]. Our research revealed distinct differential expression patterns of 17 *PYL_EUP* genes (excluding *PYL16_EUP*) in EUP across different tissues: *PYL1*/*2*/*6*/*9*/*11*/*13_EUP* was significantly enriched in roots, *PYL5*/*7*/*12*/*17_EUP* showed specifically high expression levels in leaves, and *PYL1*/*4*/*10*/*14_EUP* exhibiting predominant expression in stems.

Phylogenetic analysis of *PYL*_*EUP* genes and their *Arabidopsis thaliana* homologs revealed that *PYL1*_*EUP*, *PYL6*_*EUP*, *PYL7*_*EUP*, *PYL9*_*EUP*, *PYL13*_*EUP,* and *PYL17*_*EUP* were orthologous to *AtPYL9*, *AtPYL5*, *AtPYR1*, *AtPYL1*, *AtPYL8,* and *AtPYL4*, respectively [51]. Functional studies have demonstrated that *AtPYL9* overexpression accelerated senescence and death in older leaves while promoting summer dormancy-like responses in young tissues under severe water deficit [71]; *AtPYL4* overexpression significantly enhanced drought resistance [36]; *AtPYL8* regulated lateral root growth, with its mutation causing ABA insensitivity in roots during stress recovery [25]; and *AtPYL5* plays essential roles in stomatal responses to ABA and environmental signals by integrating various stimuli to balance water loss and CO_2_ uptake [24]. Our transcriptomic and qPCR analyses revealed that *PYL*_*EUP* genes exhibited tissue-specific induction under drought stress. Specifically, *PYL1*_*EUP* was upregulated in roots, stems, and leaves, whereas *PYL7*_*EUP* and *PYL13*_*EUP* showed leaf-specific induction. In contrast, *PYL4*/*12*/*13*_*EUP* exhibited root-specific upregulation. These results suggested that *PYL*_*EUP* genes participated in the drought response of *P. euphratica* through tissue-specific regulatory mechanisms. Notably, *PYL1*_*EUP* appeared to function as a master regulator coordinating whole-plant responses, while other members executed tissue-specific functions, collectively establishing a robust drought response system.

## 4. Materials and Methods

### 4.1. Plant Materials and Growth Conditions

This experiment selected *P. euphratica* seeds from the Awati County *P. euphratica* Forest in Xinjiang (located at the northwestern edge of the Tarim Basin, characterized by a warm-temperate extreme continental arid desert climate). Mature capsules were collected in September 2022, followed by natural drying under indoor conditions, separation of pappus from seeds, and storage in sealed brown bottles at 4 °C for later use. The seeds were uniformly sown in nutrient soil trays and cultivated in a greenhouse at 30 °C, 50% humidity, and under a 16 h light/8 h dark photoperiod for two months. Subsequently, the seedlings were transplanted to larger trays, with one seedling retained per tray for continued growth. A completely randomized design was adopted, using one-year-old *P. euphratica* seedlings as experimental material for drought treatment. Sixty healthy seedlings with uniform growth and no pests or diseases were selected and evenly divided into two groups: one subjected to drought stress and the other as a well-watered control group, with three biological replicates per group. After treatment, both groups were maintained under identical growth conditions. When leaf wilting occurred and the soil moisture content reached 21.5%, root, stem, and leaf tissues from seedlings of both groups were collected on the 13th day, immediately frozen in liquid nitrogen, and stored at −80 °C for subsequent transcriptomic analysis.

### 4.2. Data Acquisition

The genomic sequences, genome annotation files, protein sequences, and bioinformatics data of PSE, WUA, SZE, YUN, KOR, SIM, LAS, DAV, ROT, ALB, QIO, and ADE were obtained from the China National Center for Bioinformation (CNCB) (https://bigd.big.ac.cn/gwh (accessed on 8 April 2025)). The genomic data of TRI and DEL were sourced from https://phytozome-next.jgi.doe.gov/ (accessed on 8 April 2025). The genomic data of TRE were obtained from http://popgenie.org (accessed on 8 April 2025). The genomic data of PRU were sourced from Figshare (https://figshare.com/articles/online_resource/Pprgenome_fa/20705107/2 (accessed on 8 April 2025)). The telomere-to-telomere (T2T) complete genome assembly of *Populus euphratica* used in this study was independently generated by our research group and has not been publicly released. The genomic and proteomic data of *Arabidopsis thaliana* PYL family members were retrieved from the TAIR database (https://www.arabidopsis.org/ (accessed on 10 April 2025)).

### 4.3. Identification of PopPYL Genes in Populus Species

In this study, two complementary approaches were employed to systematically identify *PYL* homologs. First, the BLASTP algorithm was utilized to search the protein databases of *Arabidopsis thaliana* and 17 *Populus* species, using 13 *Arabidopsis* PYL protein sequences as queries. The E-value threshold was set to 1e^−5^ to ensure stringent homology detection. Second, an HMM-based search was conducted by scanning the same protein datasets against the PYL-specific domain model (PF10604.hmm, retrieved from the Pfam database, https://www.ebi.ac.uk/interpro/ (accessed on 11 April 2025)) [72]. The union of candidate genes obtained from both methods was then subjected to further validation. SMART v8.0 (http://smart.embl-heidelberg.de/ (accessed on 11 April 2025)) and CD-Search v3.20 (https://www.ncbi.nlm.nih.gov/Structure/ (accessed on 11 April 2025)) were used to confirm the presence of complete PYL conserved domains, eliminating false-positive hits. The final high-confidence gene list was compiled, and TBtools was employed for batch calculation of molecular weights and isoelectric points (pI) of the identified PYL proteins.

### 4.4. Phylogenetic Analysis of PopPYL Family Protein Sequences

Multiple sequence alignment of *PopPYL* family protein amino acid sequences was conducted using the MUSCLE algorithm implemented in MEGA 7.0 software. Based on the alignment results, a phylogenetic tree was reconstructed through the Neighbor-Joining (NJ) method with 1000 bootstrap replicates to evaluate nodal support reliability. The final phylogenetic tree (Newick format: *.nwk) was subsequently visualized and annotated using the iTOL v6 online platform (http://itol.embl.de/ (accessed on 12 April 2025)).

### 4.5. Motif Analysis and Gene Structure

The conserved motifs in PopPYL proteins were predicted using the MEME Suite v 5.5.5 (https://memesuite.org/meme/tools/meme (accessed on 14 April 2025)) with parameters set to 10 motifs per sequence and an E-value cutoff < 1 × 10^−5^ [73]. Functional domain annotation was performed using NCBI’s Batch CD-Search v3.20 tool (https://www.ncbi.nlm.nih.gov/Structure/bwrpsb/bwrpsb.cgi (accessed on 14 April 2025)) with default threshold settings. The predicted motifs and annotated domains were integrated and visualized using TBtools software (v2.309) to generate comprehensive schematic representations.

### 4.6. Gene Duplication Analysis and Ka/Ks Calculation

Gene Duplication Analysis: Whole-genome synteny analysis of *PYL* genes was conducted among EUP, PRU, TRI, and ADE lineages using TBtools software. Homologous gene pairs were identified through whole-genome alignment with the “MCScanX” module in TBtools, based on species-specific genome annotation files and genomic sequences.

*Ka*/*Ks* Analysis: Evolutionary selection pressure was assessed by calculating Ka and Ks values with *Ka*/*Ks* ratios using *Ka*/*Ks*_Calculator v2.0 [74]. Generally, *Ka*/*Ks* = 1 indicates neutral mutation, *Ka*/*Ks* > 1 suggests positive selection, and *Ka*/*Ks* < 1 represents purifying selection.

### 4.7. SVs and Gene Expression Analysis of PYL_EUP

This study constructed a pan-genome of 17 *Populus* species using the *P. euphratica* (EUP) reference genome and performed structural variant (SV) analysis, generating a novel SV dataset (17genome.sv.vcf). Functional annotation of SVs was conducted with ANNOVAR (2018Apr16) to extract key VCF features including chromosomal coordinates, variant boundaries, and reference/alternative alleles. Through custom scripts, we characterized SV distributions in genic regions (coding sequences, introns) and flanking regulatory regions (±2 kb). SVs associated with the *PYL_EUP* gene family were filtered from the annotated results. Gene expression profiles across all 17 *Populus* samples were obtained from the PSIR database (http://www.populus-superpangenome.com/ (accessed on 20 April 2025)). The Mann–Whitney U test was systematically employed to assess the association between SV presence/absence patterns and *PYL* gene expression levels. *PYL* genes with *p*-values < 0.05 were classified as showing SV-associated expression alterations.

### 4.8. Analysis of Promoter Regions of PopPYL Genes

The 2000 bp upstream sequences from the translation start sites of *PYL_EUP* and *PYL_PRU* genes were extracted using TBtools (v2.309). These promoter sequences were then analyzed in the PlantCARE database (http://bioinformatics.psb.ugent.be/webtools/plantcare/html/ (accessed on 23 April 2025)) to predict putative cis-regulatory elements. The identified cis-acting elements were visualized using TBtools (v2.309) [75].

### 4.9. Analysis of Gene Expression Profiles

Differential gene expression analysis between sample groups was conducted using DESeq2 to identify differentially expressed genes (DEGs) under two biological conditions [76]. The obtained P-values were adjusted for multiple testing using the Benjamini–Hochberg method to control the false discovery rate (FDR). DEGs were filtered with thresholds of |log_2_FoldChange| ≥ 1 and FDR < 0.05. Expression patterns of *PYL_EUP* and *PYL_PRU* genes were visualized through heatmaps generated with TBtools software (v2.309).

### 4.10. qRT-PCR Validation 

To further validate the accuracy of the sequencing data, we selected six significantly differentially expressed genes (DEGs) in different tissues of *P. euphratica* for qRT-PCR verification. Gene-specific primers for qRT-PCR were designed using the NCBI Primer-BLAST tool v6.25 (https://www.ncbi.nlm.nih.gov/tools/primer-blast/ (accessed on 25 April 2025)) (Table S5). Total RNA was extracted using the Total RNA Extractor (Trizol) Plant RNA Kit (B511311, Sangon Biotech (Shanghai, China)). Genomic DNA contamination was evaluated by 1.5% agarose gel electrophoresis, and RNA concentration and OD values were determined. First-strand cDNA was synthesized using Maxima Reverse Transcriptase (EP0743, Thermo Fisher Scientific). qPCR amplification was performed on an ABI 7500 Real-Time PCR System (Thermo Fisher Scientific, Waltham, MA, USA) in 20 μL reaction volumes containing 10 μL SGExcel FastSYBR Mixtures, 0.4 μL each of forward and reverse primers (10 μM), 7.2 μL RNase-Free ddH_2_O, and 2 μL cDNA template. The thermal cycling conditions consisted of initial denaturation at 95 °C for 3 min, followed by 45 cycles of 95 °C for 15 sec and 60 °C for 45 sec (annealing/extension). Three biological and technical replicates were performed for each sample.

## 5. Conclusions

A pan-genomic analysis of 17 *Populus* species identified 30 *PopPYL* pan-genes exhibiting significant copy number variation, representing 325 distinct *PYL* gene homologues. These findings indicate frequent gene duplication and loss events during *Populus* evolution. Phylogenetic classification divided *PopPYL* into three clades (I–III), where clade III was the most expanded (21 members) and clade I the most conserved (four members). Species-specific distribution patterns were observed for *PYL3* and *PYL8*, while the absence of *PYL15* in EUP and PRU, and the unique acquisition of *PYL23*, reflected functional remodeling during adaptive evolution. Gene structure and conserved motif analyses revealed similar exon–intron architectures and motif compositions within subfamilies, although distinctive SVs in *PYL7_TRE*/*PRU* indicated potential neofunctionalization. Synteny and selection pressure analyses identified 61 homologous gene pairs resulting from whole-genome duplication events, and *PYL4*/*6*/*7*/*9* showed positive selection (*Ka*/*Ks* > 1) across multiple species, consistent with their roles in drought response. Promoter analysis revealed complex regulatory networks, featuring abundant MYB elements in *PYL9_EUP*/*PRU*, enriched ARE elements in *PYL13_EUP,* and the highest ABRE content in *PYL17_PRU*, providing a basis for tissue-specific expression. SV analysis detected six SV loci significantly affecting *PYL* gene expression patterns (*p* < 0.05). Expression profiling confirmed distinct functional specialization: root-enriched *PYL1*/*6*/*13_EUP* likely regulated root-cap ABA signaling; leaf-specific *PYL7*/*17_EUP* might participate in stomatal regulation; while stem-predominant *PYL1*/*4*/*10_EUP* potentially coordinated long-distance ABA transport. Crucially, *PYL1_EUP* showed drought-induced upregulation in roots, stems, and leaves, and *PYL1* and *PYL13* exhibited high expression levels across detection methods, demonstrating their roles as core regulatory nodes in poplar drought resistance. These findings advance our understanding of the biological functions of *PYL* genes and provide a foundation for breeding stress-resistant poplar varieties.

## Figures and Tables

**Table 1:** Statistics of data sources.

Species	Abbreviation	Assembly Size (Mb)	Genome Data	Reference
*Populus euphratica*	EUP	518.6	Unpublished	
*Populus pruinosa*	PRU	521.1	https://www.ncbi.nlm.nih.gov/search/all/?term=PRJNA863418Pprgenome_fa/20705107/2	[43]
*Populus pseudoglauca*	PSE	448.7	Database: https://bigd.big.ac.cn/gwh; Accession number: GWHBJUF00000000	[39]
*Populus wuana*	WUA	417.4	Database: https://bigd.big.ac.cn/gwh; Accession number: GWHBJUE00000000	[39]
*Populus szechuanica*	SZE	429.1	Database: https://bigd.big.ac.cn/gwh; Accession number: GWHBJUK00000000	[39]
*Populus yunnanensis*	YUN	433.7	Database: https://bigd.big.ac.cn/gwh; Accession number: GWHBJUD00000000	[39]
*Populus koreana*	KOR	401.4	Database: https://bigd.big.ac.cn/gwh; Accession number: GWHBHRS00000000	[44]
*Populus trichocarpa v4.1*	TRI	392.2	Database: https://phytozome-next.jgi.doe.gov/	Phytozome
*Populus deltoids v2.1*	DEL	446.8	Database: https://phytozome-next.jgi.doe.gov/	Phytozome
*Populus simonii*	SIM	408.0	Database: https://bigd.big.ac.cn/gwh; Accession number: GWHBJTX00000000	[39]
*Populus lasiocarpa*	LAS	419.5	Database: https://bigd.big.ac.cn/gwh; Accession number: GWHBJUB00000000	[45]
*Populus davidiana*	DAV	441.1	Database: https://bigd.big.ac.cn/gwh; Accession number: GWHBJTW00000000	[46]
*Populus rotundifolia*	ROT	414.3	Database: https://bigd.big.ac.cn/gwh; Accession number: GWHBJUA00000000	[39]
*Populus tremula*	TRE	408.8	Database: http://popgenie.org	[47]
*Populus alba var. pyramidalis*	ALB	408.1	Database: http://bigd.big.ac.cn/bioproject; Accession number: PRJCA002423	[48]
*Populus qiongdaoensis*	QIO	391.3	Database: http://bigd.big.ac.cn/bioproject; Accession number: PRJCA007862	[49]
*Populus adenopoda*	ADE	383.4	Database: https://bigd.big.ac.cn/gwh; Accession number: GWHBJTV00000000	[50]

Note: URL (Accessed on: 8 April 2025).

**Table 2:** Summary of PYL protein properties.

	Sequence ID	Number of Amino Acid	Molecular Weight	Theoretical pI	Instability Index	Aliphatic Index
I	PYL1	54~189	9673.47~21,213.94	5.89~10.17	38.12~43.1	81.9~110
	PYL2	190~205	21,377.46~2,3149.56	5.6~7.73	34.04~39.96	88.53~91.63
	PYL3	706	77,272.84	5.22	30.48	85.44
	PYL4	169~248	18,686~27,574.39	4.44~5.15	26.15~44.62	66.73~77.65
	PYL24	202	22,653.53	4.7	47.65	74.75
	PYL14	168~381	18,895.38~43,039.84	4.46~4.8	25.8~31.73	72.66~78.99
	PYL21	188	21,413.6	8.44	34.03	81.38
	PYL19	108~143	12,205.97~16,086.06	4.39~4.59	40.25~43.88	76.36~92.96
	PYL15	169~329	18,665.8~36,424.99	4.71~5.55	29.9~42.91	72.12~86.8
	PYL18	102~115	11,253.82~12,760.6	5.57~5.86	26.7~36.85	77.91~85
	PYL12	103~163	11,430.91~18,163.62	4.46~4.9	23.22~45.19	77.04~93.74
	PYL29	163	18,053.43	4.64	29.94	86.01
	PYL26	186	20,986.81	6.44	39.15	84.25
	PYL8	615	67,740.22	7.23	38.38	93.07
	PYL30	190	21,527.63	6.06	38.55	91.63
	PYL25	190	21,487.52	6.06	40.01	89.05
	PYL27	190	21,463.46	6.06	40.31	88.53
	PYL23	220	24,606.14	5.62	43.17	92
	PYL13	191~229	21,355.14~25,681.5	5.78~6.11	40.64~48.96	94.29~98.25
	PYL28	191	21,369.16	5.78	48.96	94.29
	PYL16	134~282	15,264.5~32,181.38	5.55~8.03	38.94~44.91	95.9~100.9
II	PYL6	186~223	20,570.31~24,181.23	8.5~9.21	42.26~46.91	80.59~85.02
	PYL17	214~222	23,268.13~24,100.99	7.66~8.26	50.16~54.14	79.41~80.56
	PYL22	306	34,088.89	6.3	46.78	93.24
	PYL10	214~238	23,103.88~26,319.76	7.11~9.22	38.54~51.13	78.35~85.47
	PYL11	214	23,086.16~23,310.38	7.1~7.68	49.26~54.69	86.82~90.93
III	PYL5	124~191	13,517.37~21,341.06	5.2~7.01	2.05~51.39	82.43~89.69
	PYL9	203~210	22,319.97~23,190	4.93~5.53	32.74~42.8	82.1~86.85
	PYL7	201~259	22,392.17~28,658.25	5.08~5.62	29.23~34.58	79.57~84.33
	PYL20	169	18,961.56~18,961.58	5.94	38.88~40.02	85.27~85.86

## Data Availability

Data are contained within the article and Supplementary Materials.

## Supplementary Materials

The following supporting information can be downloaded at https://www.mdpi.com/article/10.3390/plants14162541/s1, Table S1. Identification of *PYL* genes in the 17 *Populors*; Table S2. Pan-gene list of the 17 *Populors*; Table S3. PYL gene family of *Populus euphratica* (EUP); Table S4. RNA-seq data of *PYL_EUP*; Table S5. Primer sequences.
